# Favourable HDL composition in endurance athletes is not associated with changes in HDL *in vitro* antioxidant and endothelial anti-inflammatory function

**DOI:** 10.1042/BSR20241165

**Published:** 2024-10-23

**Authors:** Jack David Beazer, Anne Sillars, Sally Beck, Christina Christoffersen, Maria J. Ferraz, Monique T. Mulder, Delyth Graham, Helen Karlsson, Stefan Ljunggren, Jason Gill, Dilys J. Freeman

**Affiliations:** 1 School of Cardiovascular and Metabolic Health, Glasgow Cardiovascular Research Centre, University of Glasgow, 126 University Place, Glasgow, G12 8TA, United Kingdom; 2 Department of Clinical Biochemistry, Section 3-01-3, Rigshospitalet, Blegdamsvej 9, 2100 Copenhagen, Denmark and Institute of Biomedical Sciences, University of Copenhagen, Blegdamsvej 3A, 2200 Copenhagen, Denmark; 3 Medical Biochemistry, Leiden Institute of Chemistry, Leiden University, Gorlaeus Building, Einsteinweg 55, 2333 CC Leiden, The Netherlands; 4 Division of Pharmacology, Vascular and Metabolic Diseases, Department of Internal Medicine, Erasmus University Medical Centre, Dr Molewaterplein 40, 3015 GD Rotterdam, The Netherlands; 5 Occupational and Environmental Medicine Center in Linköping, Department of Health, Medicine and Caring Sciences, Linköping University, SE-58183 Linköping, Sweden

**Keywords:** endothelial function, exercise, high-density lipoprotein, insulin resistance

## Abstract

Given the failure of high-density lipoprotein (HDL) raising therapies to reduce cardiovascular disease risk, attention has turned towards HDL composition and vascular protective functions. In individuals with insulin resistance, exercise interventions recover HDL function. However, the effect of exercise on HDL in otherwise healthy individuals is unknown. This cross-sectional study aimed to measure HDL composition and antioxidant/endothelial anti-inflammatory function in insulin sensitive endurance athlete and healthy control men. HDL was isolated using density gradient ultracentrifugation. HDL composition was measured using microplate assays for apolipoprotein A-I, total cholesterol content and apolipoprotein M. HDL protein composition was measured using nano-liquid chromatography tandem mass spectrometry. HDL subclass distribution was measured by native gel electrophoresis. HDL *in vitro* antioxidant function was measured by paraoxonase-1 activity assay and anti-inflammatory function assessed in endothelial cells. Compared with controls, endurance athlete HDL had higher apolipoprotein A-1 (1.65 ± 0.62 mg/ml vs 1.21 ± 0.34 mg/ml, *P*=0.028) and higher total cholesterol content (2.09 ± 0.44 mmol/L vs 1.54 ± 0.33 mmol/L, *P*<0.001). Proteomics revealed higher apolipoprotein A-II, A-IV and D and transthyretin in endurance athlete HDL versus controls. There was no difference observed in *in vitro* HDL antioxidant or anti-inflammatory functions between controls and endurance athletes. Despite a more favourable composition, endurance athlete HDL did not have higher *in vitro* antioxidant or anti-inflammatory function. It is possible that HDL has a ceiling of function, i.e. that healthy HDL function cannot be enhanced by endurance exercise.

## Introduction

Plasma high-density lipoprotein (HDL) cholesterol (HDL-C) concentration was first established as an inverse predictor of cardiovascular disease (CVD) risk during the Framingham Heart Study in the 1970s, an effect proposed to be mediated via reverse cholesterol transport [1,2]. However, though pharmacological interventions to inhibit cholesteryl ester transfer protein (CETP) were successful at increasing plasma HDL cholesterol, they did not modify cardiovascular risk [3–6]. Attention therefore turned towards HDL composition and function, rather than plasma concentration, as a mediator of cardiovascular risk [7].

Conditions of insulin resistance such as obesity, metabolic syndrome and Type 2 diabetes (T2DM) have a number of detrimental effects on HDL composition and function. Obesity is associated with reduced HDL concentration, apolipoprotein (apo) A-I content and proportion of large buoyant HDL 2 particles [8,9]. HDL paraoxonase 1 (PON-1) activity was lower in overweight and obese individuals [10]. Obese individuals with the metabolic syndrome had altered HDL apolipoprotein and sphingosine-1-phosphate (S1P) content, and their HDL had reduced anti-inflammatory and antioxidant function [11,12]. In T2DM, HDL had altered composition including higher serum amyloid protein A (SAA) content and reduced HDL- CEC, antioxidant and anti-inflammatory function [13,14]. Furthermore, HDL PON-1 activity was a stronger predictor of the incidence of coronary artery disease in T2DM than plasma HDL cholesterol [15].

The effect of exercise on metabolic health has been well documented. A meta-analysis of 160 randomised controlled trials found that exercise increased cardiorespiratory fitness, improved lipid profile (particularly increased HDL-C) and improved markers of glucose/insulin metabolism. It is notable that the beneficial effects were most marked in people aged <50 years, men, and people with metabolic diseases [16]. Exercise also alters HDL composition and improves HDL function in individuals with insulin resistance. Four months of aerobic exercise training in individuals with T2DM increased HDL size and HDL-CEC [17]. Three months of bicycle ergometer training in individuals with metabolic syndrome increased HDL PON-1 activity [18]. Despite no increase in HDL cholesterol concentration, 10-weeks of walk-run training in a similar cohort increased HDL PON-1 activity, improved HDL-mediated inhibition of adhesion molecule expression and monocyte recruitment in endothelial cells and prevented TNFα inhibition of eNOS-mediated nitric oxide generation [19]. This evidence combines to suggest that insulin resistance reduces HDL function and that interventions to improve insulin resistance can improve HDL function from its impaired state.

There are far fewer data on the impact of exercise on HDL composition and function in healthy individuals. It is important to understand whether HDL composition can be altered, and function improved, beyond that observed in healthy sedentary individuals or whether improvements can only be observed in those with an *a priori* insulin resistance-mediated impairment in HDL function. The hypothesis of the present study was that there is altered HDL composition with a positive impact on HDL function in middle-aged endurance athletes compared with age-matched healthy sedentary controls. The aim of this cross-sectional study was to assess HDL composition and function in a group of middle-aged men with high insulin sensitivity i.e. endurance athletes and compare them with healthy controls. A comparator group of middle-aged men with impaired glucose regulation, where HDL function was expected to be reduced, was also included as a well-characterised positive control. HDL compositional analysis, including determination of the HDL proteome, was carried out in each group and HDL *in vitro* vascular function was assessed in a cell-based anti-inflammation assay.

## Materials and methods

### Participant recruitment

Participants were originally recruited to a previous study of muscle triglyceride content and insulin resistance [20]. The study was carried out in accordance with the World Medical Association Declaration of Helsinki and all participants provided written informed consent. The present study made use of all the recruited participants. The study had 80% power at the 5% significance level to detect a 1 standard deviation difference in intramuscular triglyceride between the groups. Briefly, lean normoglycemic men who did not participate in regular vigorous exercise (normal insulin sensitivity, *n*=18), men with impaired glucose regulation (IGR) (low insulin sensitivity, *n*=17) and endurance athletes undertaking >5 h per week of vigorous exercise for at least 2 years (high insulin sensitivity, *n*=20) underwent a 2-h oral glucose tolerance test after an overnight fast, a maximal incremental cycle ergometer test to assess maximal oxygen uptake and a muscle and adipose tissue biopsy. Normoglycemic and endurance athlete men were recruited based on an age of between 30 and 60 years, a body mass index (BMI) of between 18 and 27 kg/m^2^, a glycated haemoglobin (HbA1c) of <43.8 mmol/mol and no history of vascular or metabolic disease or any medications affecting lipid or carbohydrate metabolism. Men with impaired glucose regulation were recruited in the same age range and either a HbA1c of between 43.8 and 47.4 mmol/mol, impaired fasting glucose or impaired glucose tolerance, or T2DM controlled by lifestyle only. Participants were excluded from the study if they were on any treatment for glucose control. Participants underwent a 2-h 7 5g oral glucose tolerance test (OGTT) as well as broader blood biochemistry and anthropometric measurements. Insulin resistance was calculated using fasting glucose and insulin concentrations by Homeostatic Assessment Model for Insulin Resistance (HOMA_IR_) [21] as follows:

$$HOMA_{IR}\;=\;\frac{\left(fasting\;glu\cos e\;[mmol/L]\;\times \;fasting\;insulin\;[mIU/L]\right)}{22.5}$$

Insulin sensitivity was calculated using the Matsuda Index [22], which uses glucose and insulin concentrations throughout the OGTT as follows:

Matsuda index = √[(fasting glucose × fasting insulin) × (OGTT mean glucose × OGTT mean insulin)]

### Isolation of HDL from plasma

Blood samples (20–40 ml) were collected in tubes containing ethylenediaminetetraacetic acid (EDTA). Plasma was separated from whole blood through centrifugation at 1500 × ***g*** for 15 min at 4°C and stored in aliquots at −80°C until use. HDL was isolated from 500 μl plasma by flotation in a two-step process using sodium bromide/EDTA density solutions as previously described [23]. Briefly, VLDL and LDL were first removed from plasma at a density of 1.063 g/ml. Samples were centrifuged in a Beckman Coulter TLA 120.2 rotor and Optima MAX-TL centrifuge at 100,000 RPM (355,040 × ***g*** average) for 2.5 h at 23°C. After removal of VLDL and low-density lipoprotein (LDL), HDL was isolated at a density of 1.21 g/ml. The samples were centrifuged again at 100,000 RPM (355,040 × ***g*** average) for 5 h at 23°C. Excess salt was removed from the resulting HDL fractions using PD MiniTrap G-25 columns (Cytiva, Little Chalfont, Buckinghamshire, U.K., #28918007), according to manufacturer’s instructions.

### Measurement of HDL constituents

HDL apoA-I and serum amyloid A1 (SAA-1) content was measured using enzyme linked immunosorbent assay (ELISA) kits (Duoset, R&D systems) with the requisite ancillary kits, according to manufacturer’s instruction. HDL total protein content was measured by Bradford assay [24]. HDL total cholesterol content was measured using a cholesterol quantitation kit (Sigma-Aldrich, #MAK043), according to the manufacturer’s protocol.

### Measurement of HDL subclass distribution

HDL subclass distribution was measured using native polyacrylamide gel electrophoresis (PAGE), according to [25] with some modification. Native-PAGE was performed using precast 4–20% tris-glycine gels in an XCell SureLock Mini-Cell Electrophoresis System (Thermofisher). HDL diluted in native sample buffer (40 µl) was loaded into each well of the gel. NativeMark unstained protein standard (5 µl, Thermofisher Scientific #LC0725) was used as the molecular weight marker. Electrophoresis was conducted at 225 V constant for 50 min. Gels were stained for protein in 50 ml QC Colloidal Coomassie stain (Bio-Rad Laboratories #1610803) for 1 h at room temperature, followed by de-staining in three changes of dH2O over 1 h. Gels were imaged using a LI-COR Odyssey FC imager in the 700 nm channel for 2 min. To analyse the HDL subclass distributions, the molecular weight marker was first converted to diameter (in nm) using the calculator found at ‘www.nanocomposix.com/pages/molecular-weight-to-size-calculator’ (underpinning mathematical model described elsewhere [26]). The standardised retention factor (RF) for each marker band was calculated relative to the 66 kDa marker band and a linear standard curve drawn. This standard curve was used to calculate the maximum and minimum RF values for each of the following HDL subclass based on their diameter: 2b 9.7–12.9 nm, 2a 8.8–9.7 nm, 3a 8.2–8.8 nm, 3b 7.8–8.2 nm, 3c 7.2–7.8 nm [27]. The background adjusted signal for each HDL subclass was expressed as percentage of the total background adjusted HDL signal.

### Proteomic analysis of HDL fractions

Proteomic analyses were performed, according to [28] with some modifications. A previous study [29] found differences in the proteomic composition of HDL in a model of endotoxemia using an n of 10 per group. All 55 samples were used in the present proteomic analysis. HDL proteins (10 μg) were reduced by adding 2 µl of 25 mM dithiothreitol and incubating for 1 h at 60°C. Samples were subsequently alkylated by adding 2 µl of 75 mM iodoacetamide and incubating for 15 min at RT. Proteins were digested by incubating with trypsin (1:25) overnight at 37°C. Peptides were desalted using C18 ZipTip (Merck Millipore), lyophilized and reconstituted in 0.1% formic acid. Peptide concentrations were determined using a Nanodrop 1000 system (Thermo Fisher Scientific).

A total of 200 ng peptides were separated on a 20 cm EASY-Spray C18 connected to an EASY-nLC 1200 (Thermo Scientific, Waltham, MA, U.S.A.) using a linear gradient of 0.1% formic acid in water (A) and 0.1% formic acid in 80% acetonitrile (B) (7–40% B over 75 min followed by 40–100% B over 13 min and 2 min of holding at 100% B). Automated online analyses were performed with a QExactive HF mass spectrometer (Thermo Scientific) with a nano-electrospray source and a top10 data-dependent method. Raw files were searched using MaxQuant v.2.0.2.0 (Max Planck Institute of Biochemistry, Martinsried, Germany) against a Uniprot Human database (downloaded 9 February 2022) with the following parameters: trypsin was used as digestion enzyme; maximum number of missed cleavages 2; fragment ion mass tolerance 0.50 Da; parent ion mass tolerance 5.0 ppm; fixed modification – carbamidomethylation of cysteine; variable modifications – N-terminal acetylation and methionine oxidation proteins with at least two peptides, of which one was unique, and identified in at least 50% of the samples in an individual group were included in further analysis. Label-free quantification (LFQ) was done using the built-in LFQ algorithm.

### Measurement of HDL apolipoprotein M and sphingosine-1-phosphate content

HDL apolipoprotein M (apoM) content was measured using an ELISA as described in [30]. HDL S1P content was measured as previously described [31], with ^13^C_5_-S1P used as the internal standard.

### HDL paraoxonase-1 activity assay

HDL PON-1 activity was measured by monitoring the conversion of phenyl acetate to phenol and acetate in a UV visible spectrophotometer as described previously [32].

### HDL anti-inflammatory function in endothelial cells

Human microvascular endothelial cells (HMEC-1, ATCC-CRL-3243, LGC Standards, Middlesex, U.K.) were selected for this work and cultured to supplier protocols in MCDB131 media supplemented with 10 ng/ml epidermal growth factor (R&D Systems, Abingdon, Oxford, U.K., #236-EG), 1 µg/ml hydrocortisone (Tocris, Abingdon, Oxford, U.K., #4093), 10 mM L-glutamine (Sigma-Aldrich, G7513) and 10% fetal bovine serum (FBS, Sigma-Aldrich, #F9665). Briefly, HMEC-1 were preincubated with HDL (based on 300 µg/ml apoA-I) for four hours before the addition of 5 ng/ml tumour necrosis factor alpha (TNFα) for 24 h. Cells were lysed in RIPA buffer and western blotting performed to detect vascular cell adhesion molecule 1 (VCAM-1). Results are expressed as % inhibition of VCAM-1 expressed by cells treated with TNFα alone. Each culture plate contained untreated and TNFα only treated cells; samples were corrected for baseline VCAM-1 expression and normalised to the TNFα only control present on the same plate. Total protein normalisation was used as a loading control and all blots contained recombinant human VCAM-1 as a positive control. Immunodetection of VCAM-1 used a mouse anti-human VCAM-1 antibody from Santa Cruz Biotechnology, catalogue number sc-13160.

### Statistical analysis

#### Univariate analyses

Normal distribution of data was assessed by qualitative assessment of the QQ plot. Equality of standard deviation between groups was assessed by Brown–Forsythe test. Where data were normally distributed and standard deviations were equal across groups, comparisons were performed using one-way ANOVA with post*-*hoc Tukey test. Where standard deviations were not equal between groups, Welch’s corrected one-way ANOVA was used with post-hoc Dunnett’s T3 test. Where data were non-parametric and could not be normalised by log transformation, Kruskal–Wallis with post-hoc Dunn’s multiple comparison test was used. The gradient gel electrophoresis technique for HDL sizing and Western blotting are semi-quantitative and as such these data were considered to be non-parametric. Statistical analyses were performed using GraphPad Prism software version 9.50. Functional analyses were analysed by general linear model, ensuring normal distribution of residuals, followed by post-hoc Tukey test to allow for the inclusion of co-variates. Non-normal residual distributions were rectified by log transformation of the input data. General linear modelling was performed using Minitab software version 20.3. For all analyses, statistical significance was assumed at* P*<0.05. In large data sets such as those resulting from proteomic studies, multiple statistical comparisons increase the risk of type I error. However, corrections to probability values using the Bonferroni or Benjamini–Hochberg procedure require each observation to be independent. As one expects changes in HDL parameters to correlate with one and other, these procedures are not appropriate in this use case. Multivariate statistical analyses were therefore used instead to confirm the findings of the univariate analyses.

#### Multivariate analyses

Multivariate data analysis was performed on unit variance (UV) scaled demographics and protein LFQ-intensity data in SIMCA (v18.0, Sartorius Stedim Data Analytics AB, Umeå, Sweden) similarly to previously described [33]. Orthogonal partial least squares-discriminant analysis (OPLS-DA) was used to investigate separation of groups based on demographic, anthropometric and protein data. To find variables important for the separation of groups, a variable influence on projection (VIP) value >1.0 and VIP value > standard error (SE) were used. Resulting variables were subsequently included in a second OPLS-DA model with one predictive and one orthogonal component. Model quality was evaluated using *R*2 and *Q2* describing the goodness of fit and prediction, respectively, and ANOVA of Cross-Validated predictive residuals (CV-ANOVA).

## Results

### Group demographics

Demographic and anthropometric data for the M-FAT study participants are described in Table 1. Mean (standard deviation [SD]) ages were 53 [5], 45 (10) and 42 [6] years for IGR, control and athlete groups respectively and mean BMI and HbA1c were highest in the IGR group. Mean HDL concentration was lowest in the IGR group. Mean Matsuda index was highest in the endurance athletes (10.8 [3.6]) followed by controls (6.2 [1.6]) and IGR (2.6 [1.3], *P*<0.0001). Mean HOMA_IR_ was highest in the IGR group (5.82 [5.83]) compared with control and athlete (1.36 [0.39] and 0.99 [0.48], respectively, *P*<0.0001). Full results from the 2-h OGTT can be found in the supplemental data. The mean 10-year Framingham CVD risk score [34] for each group was 15 [7] % in men with IGR, 8 [7] % for controls and 6 [4] % for endurance athletes.

**Table 1:** Study participant characteristics

	IGR (*n*=17)	Control (*n*=18)	Athlete (*n*=20)	*P*-value
Age (years)	53 ± 5^A^	45 ± 10^B^	42 ± 6^B^	0.0001
BMI (kg/m^2^)	30.5 ± 3.4^A^	23.8 ± 1.8^B^	23.4 ± 1.5^B^	<0.0001
Body fat %	31.8 ± 7.3^A^	19.4 ± 5.5^B^	13.0 ± 5.8^C^	<0.0001
Waist/Hip ratio	1.05 ± 0.08^A^	0.93 ± 0.07^B^	0.88 ± 0.06^B^	<0.0001
Systolic blood pressure (mmHg)	142 ± 14^A^	131 ± 12^B^	135 ± 10^AB^	0.030
Diastolic blood pressure (mmHg)	90 ± 7^A^	80 ± 6^B^	79 ± 7^B^	<0.0001
VO_2_ Max (ml/kg/min)	31.0 ± 5.6^A^	35.8 ± 5.6^B^	54.7 ± 5.0^C^	<0.0001
HbA1c (mmol/mol)	53.4 ± 9.3^A^	33.1 ± 3.0^B^	34.1 ± 1.9^B^	<0.0001
Total cholesterol (mmol/l)	5.03 ± 1.02	5.02 ± 0.97	5.08 ± 1.04	0.98
Triglycerides (mmol/L)	2.46 ± 1.57^A^	1.71 ± 0.76^A^	1.13 ± 0.51^B^	0.002
LDL cholesterol (mmol/L)	2.63 ± 0.82	2.86 ± 0.88	2.93 ± 0.87	0.60
HDL cholesterol (mmol/L)	1.13 ± 0.24^A^	1.27 ± 0.26^AB^	1.47 ± 0.30^B^	0.002
Fasting insulin (µU/ml)	15.3 ± 13.7^A^	5.9 ± 1.7^B^	4.4 ± 2.0^B^	<0.0001
Fasting glucose (mmol/L)	8.20 ± 1.72^A^	5.17 ± 0.39^B^	4.93 ± 0.39^B^	<0.0001
2-h glucose (mmol/L)	13.80 ± 3.90^A^	5.04 ± 1.14^B^	4.74 ± 1.23^B^	<0.0001
Matsuda index	2.6 ± 1.3^A^	6.2 ± 1.6^B^	10.8 ± 3.6^C^	<0.0001
HOMA_IR_	5.82 ± 5.83^A^	1.36 ± 0.39^B^	0.99 ± 0.48^B^	<0.0001
10-year Framingham Risk (%)	14.6 ± 6.6^A^	8.1 ± 6.6^B^	5.5 ± 3.5^B^	<0.0001

Data expressed as mean ± SD. Comparisons were made by one-way ANOVA and post-hoc Tukey test. Means that do not share a letter are significantly statistically different. BMI, body mass index; HbA1c, glycated haemoglobin; HDL, high-density lipoprotein; HOMA_IR_, homeostatic assessment model for insulin resistance; LDL, low-density lipoprotein; VO_2_ Max, maximal oxygen consumption.

### HDL apoA-I content, cholesterol content and subclass distribution in endurance athletes, men with IGR and controls

**Figure 1:**
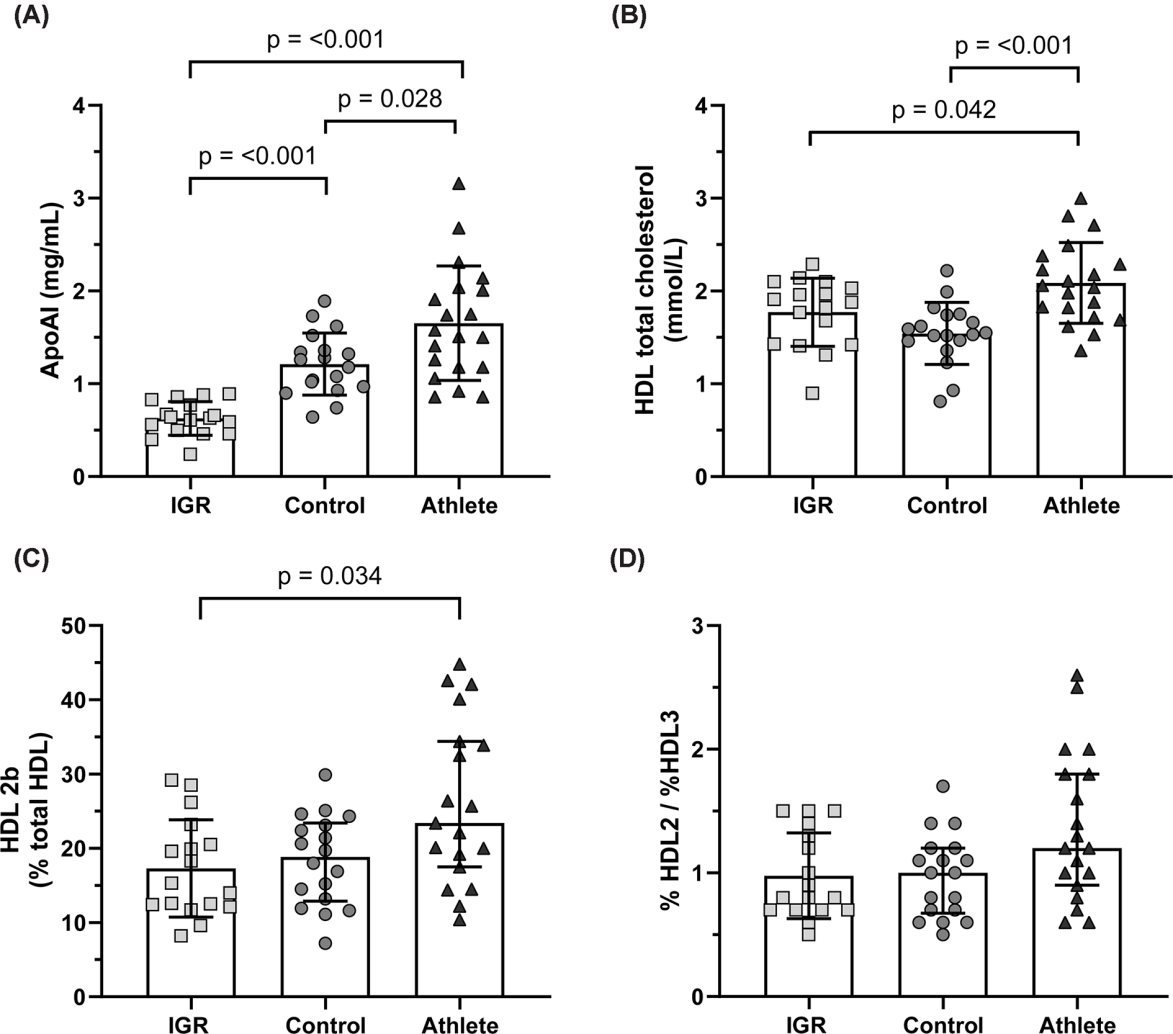
Measures of HDL constituents and subclass distribution in the M-FAT study (**A**) HDL apolipoprotein AI content as measured by ELISA. Comparisons made with Welch’s ANOVA with post-hoc Dunnett’s T3 test. (**B**) HDL total cholesterol content as measured using colorimetric assay. Comparisons made with one-way ANOVA and post-hoc Tukey test. (**C**) The proportion of HDL 2b and (**D**) the ratio of HDL 2 to HDL 3 in the MFAT study as measured using native PAGE. Comparisons made with Kruskal–Wallis test with post-hoc Dunn’s test. In all panels, data expressed as mean ± standard deviation and statistical significance assumed at *P*<0.05. ApoAI, apolipoprotein A-I; HDL, high-density lipoprotein; IGR, impaired glucose regulation.

HDL apoA-I content was significantly higher in the athlete group HDL (1.65 ± 0.62 mg/ml) compared with healthy control men (1.21 ± 0.34 mg/ml, *P*=0.028). As expected, IGR men had the lowest apoA-I content (0.63 ± 0.18 mg/ml) (Figure 1A). The total cholesterol content of HDL was significantly higher in athletes (2.09 ± 0.44 mmol/L) compared with control (1.54 ± 0.33 mmol/L) and IGR (1.77 ± 0.37 mmol/L) (Figure 1B). In terms of HDL subclass distribution, HDL 2b made up a significantly larger proportion of athlete compared with IGR HDL (26.1 ± 11.0% vs 17.3 ± 6.6%, *P*=0.034) (Figure 1C,D).

### HDL proteomic composition in endurance athletes, men with IGR and controls

#### Multivariate analysis

**Figure 2:**
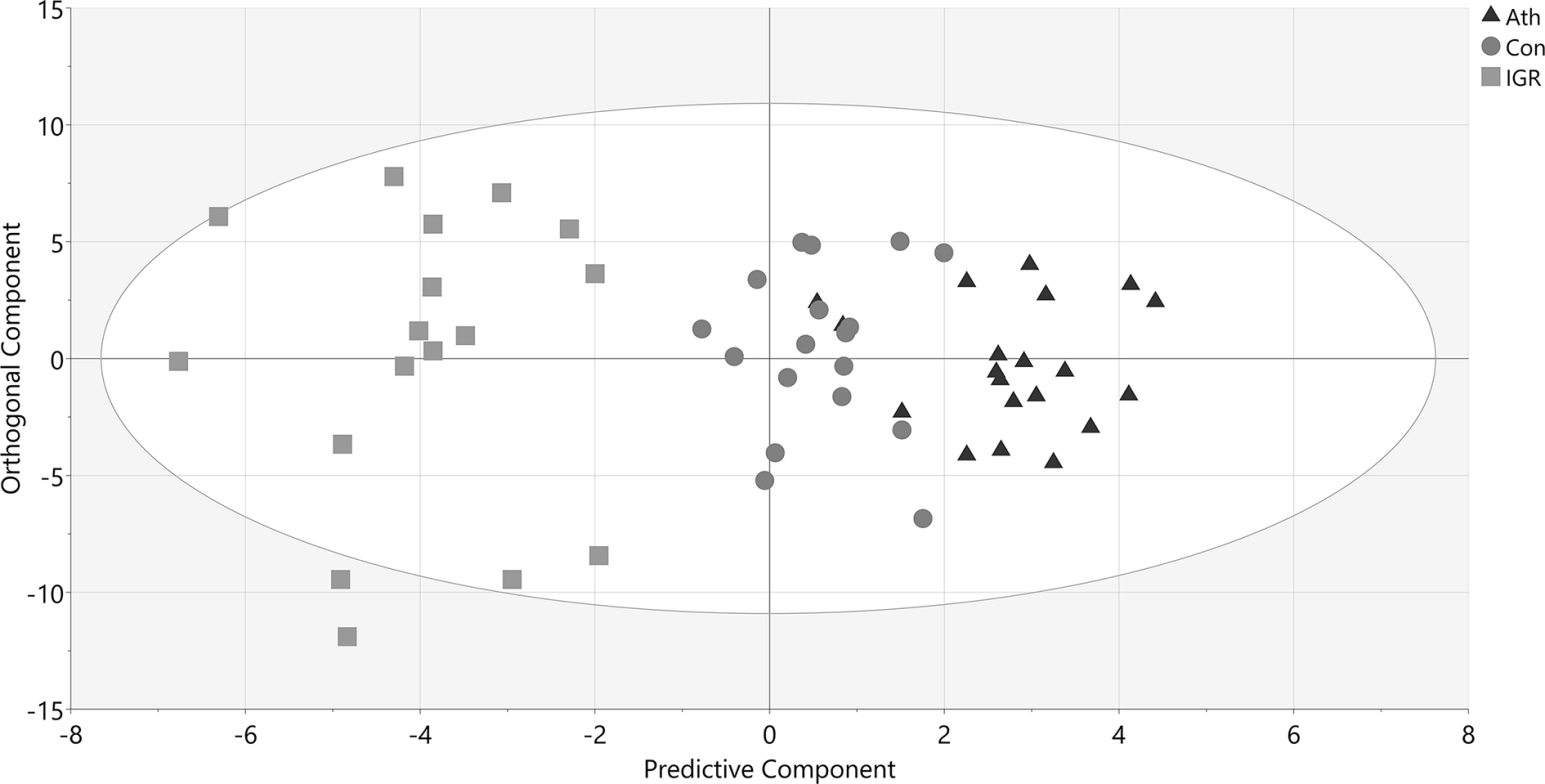
OPLS-DA model of IGR, control and athletes including demographics and identified HDL proteins Score plot of participants according to their group (triangles athletes, circles controls and squares IGR). The *x*-axis depicts the predictive component most important for separating groups, while the *y*-axis represents the orthogonal component showing in-group variation. *R*2 = 0.46, *Q*2 = 0.42, CV-ANOVA *P*-value < 0.001. Ath, endurance athlete; Con, healthy control; IGR, impaired glucose regulation; CV-ANOVA, Analysis of variance of Cross-Validated predictive residuals.

**Figure 3:**
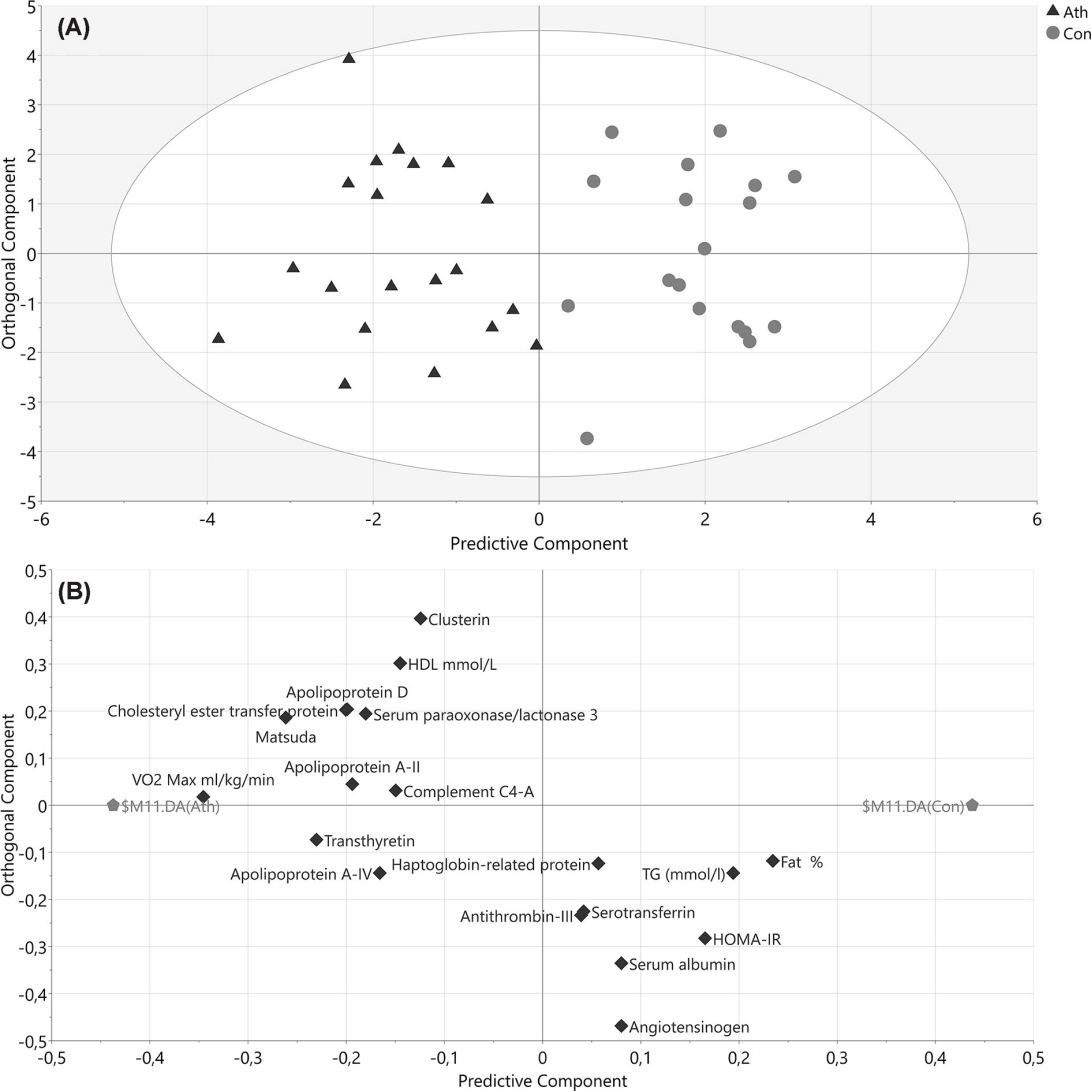
OPLS-DA model of endurance athletes and controls regarding demographics and identified HDL proteins after selecting proteins with VIP values >1.0 and VIP values >standard error (SE) (**A**) Score plot of participants according to their group (triangles athletes, circles controls). The *x*-axis depicts the predictive component most important for separating groups while the *y*-axis represents the orthogonal component showing in-group variation. *R*2 = 0.81, *Q*2 = 0.67, CV-ANOVA *P*-value <0.001. (**B**) Loading plot showing the underlying variable separation in relation to grouping. Ath, endurance athlete; Con, healthy control; CV-ANOVA, Analysis of variance of Cross-Validated predictive residuals.

**Figure 4:**
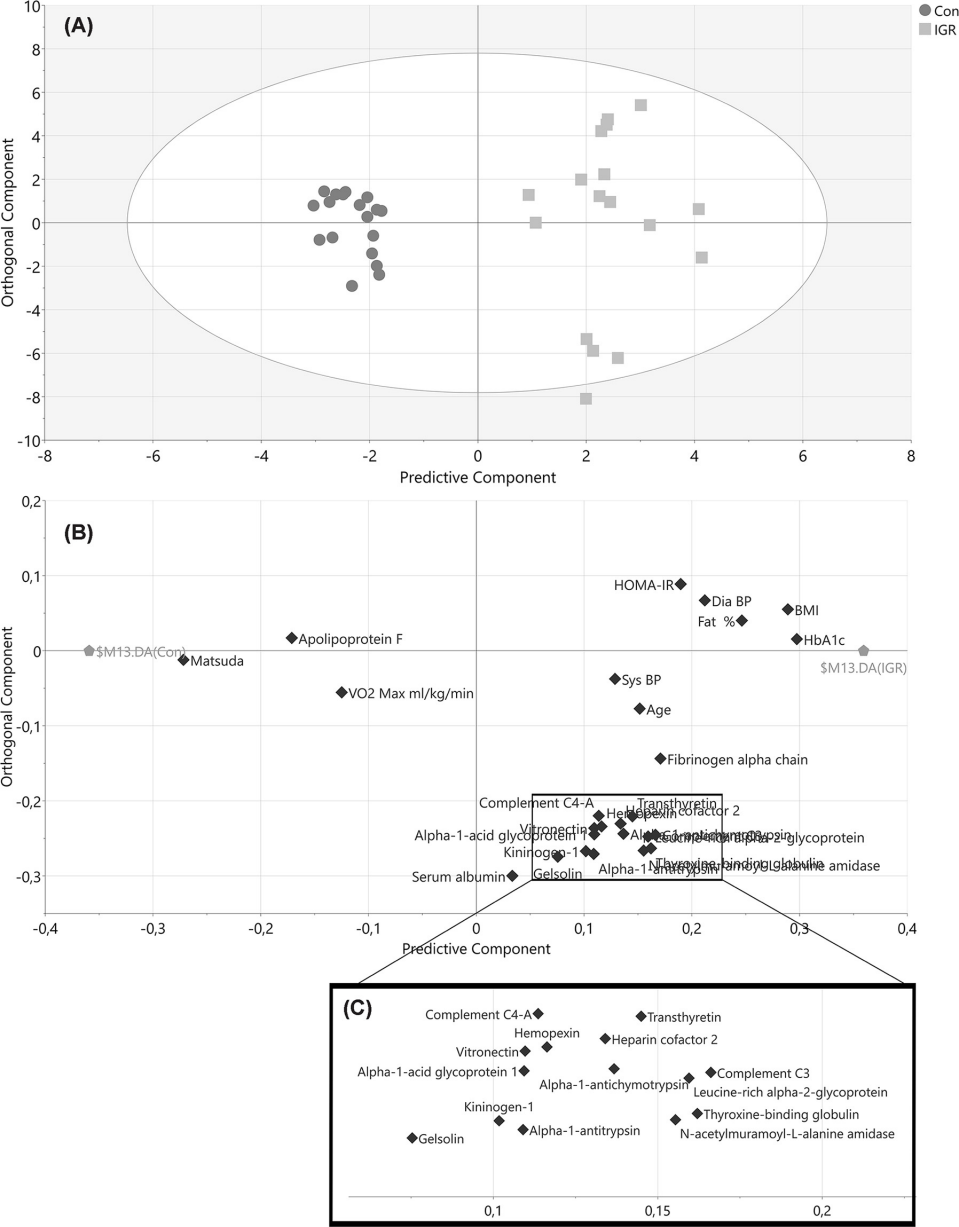
OPLS-DA model of IGR and controls regarding demographics and identified HDL proteins after selecting proteins with VIP values >1.0 and VIP values >standard error (SE) (**A**) Score plot of participants according to their group (squares IGR, circles controls). The *x*-axis depicts the predictive component most important for separating groups while the *y*-axis represent the orthogonal component showing in-group variation. *R*2 = 0.67, *Q*2 = 0.82, CV-ANOVA *P*-value = <0.001. (**B**) Loading plot showing the underlying variable separation in relation to grouping. (**C**) Inlay of box in loading plot to improve visibility of closely positioned variables. Ath, endurance athlete; IGR, impaired glucose regulation; CV-ANOVA, Analysis of variance of Cross-Validated predictive residuals.

Proteomic analysis of isolated HDL fractions by nLC-MS/MS revealed 109 proteins. Of these, 59 proteins were deemed HDL-associated based on a selection criterion of presence in >50% of HDL samples (Supplementary Data table). Protein levels were used together with demographic and anthropometric data in a multivariate model using OPLS-DA. The initial OPLS-DA model utilising all variables showed that all three groups were clearly separated (Figure 2). As expected, the IGR group was most different from controls and athletes as measured by the distance apart on the *x-*axis and had the most intra-group variation as indicated by the spread across the *y*-axis. The control and athlete groups were separated but situated in closer proximity. An OPLS-DA model comparing athletes and controls (Figure 3A) confirmed that the two groups were clearly separated. The accompanying loadings plot (Figure 3B) identified that proteins higher in athletes compared with controls included transthyretin, apoA-II, clusterin, apoD, CETP, serum paraoxonase/lactase 3, apoA-IV and complement C4-A. An OPLS-DA model comparing controls and IGR men and the accompanying loadings plot (Figure 4) indicated lower apoF in the IGR group. A number of inflammatory related proteins, including complement proteins, α-1-antitrypsin and fibrinogen, were higher in the IGR group as expected.

#### Univariate analyses

**Figure 5:**
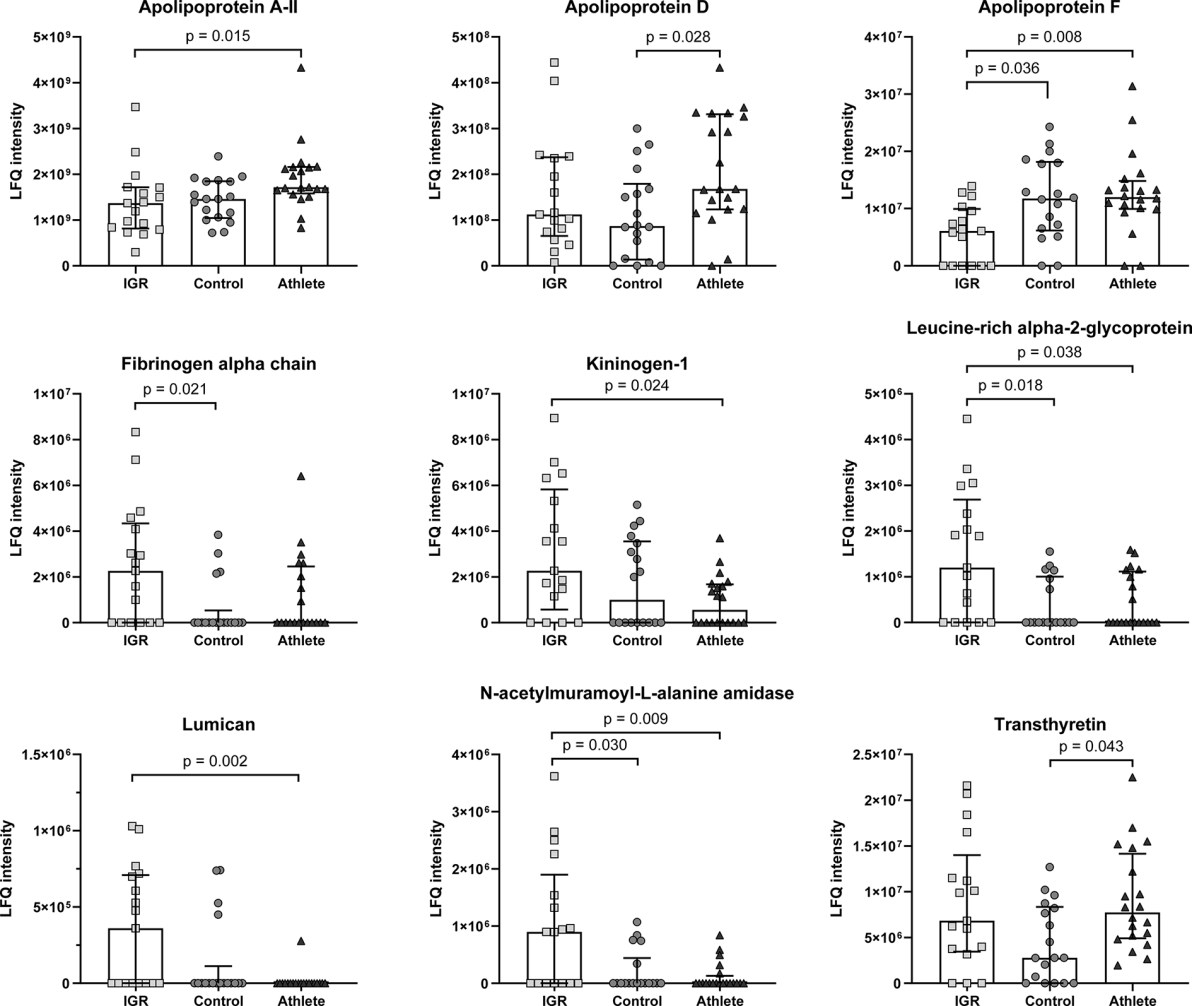
Proteins identified on HDL with significant differences between IGR, controls and athletes Comparisons made with Kruskal–Wallis test with post-hoc Dunn’s test. Data expressed as median ± interquartile range and statistical significance assumed at *P*<0.05. LFQ, label-free quantitation.

Nine proteins identified on HDL in this study had significant differences in LFQ intensity between the study groups. These included apolipoproteins, proteins involved in coagulation and proteins involved in bacterial defence, inflammation and vitamin/hormone transport (Figure 5). ApoA-II was significantly higher in the athlete compared with IGR HDL. ApoD was significantly higher in the athlete compared with control HDL. ApoF was significantly lower in the IGR HDL compared with both control and athlete HDL. Fibrinogen alpha chain was significantly higher in IGR compared with control HDL, while kininogen-1 was significantly higher in IGR versus athlete HDL. The acute phase reactant leucine-rich α-1 glycoprotein was significantly higher in IGR HDL compared with both control and athlete HDL. Lumican, a protein involved in fibrosis, was significantly higher in IGR compared with athlete HDL. The immune enzyme N-acetylmuramoyl-L-alanine amidase was significantly higher in IGR HDL compared with control and athlete HDL. The thyroxine and vitamin A transporter transthyretin was significantly higher in athlete HDL compared with control HDL.

### HDL apoM and S1P content in athletes, men with IGR and controls

**Figure 6:**
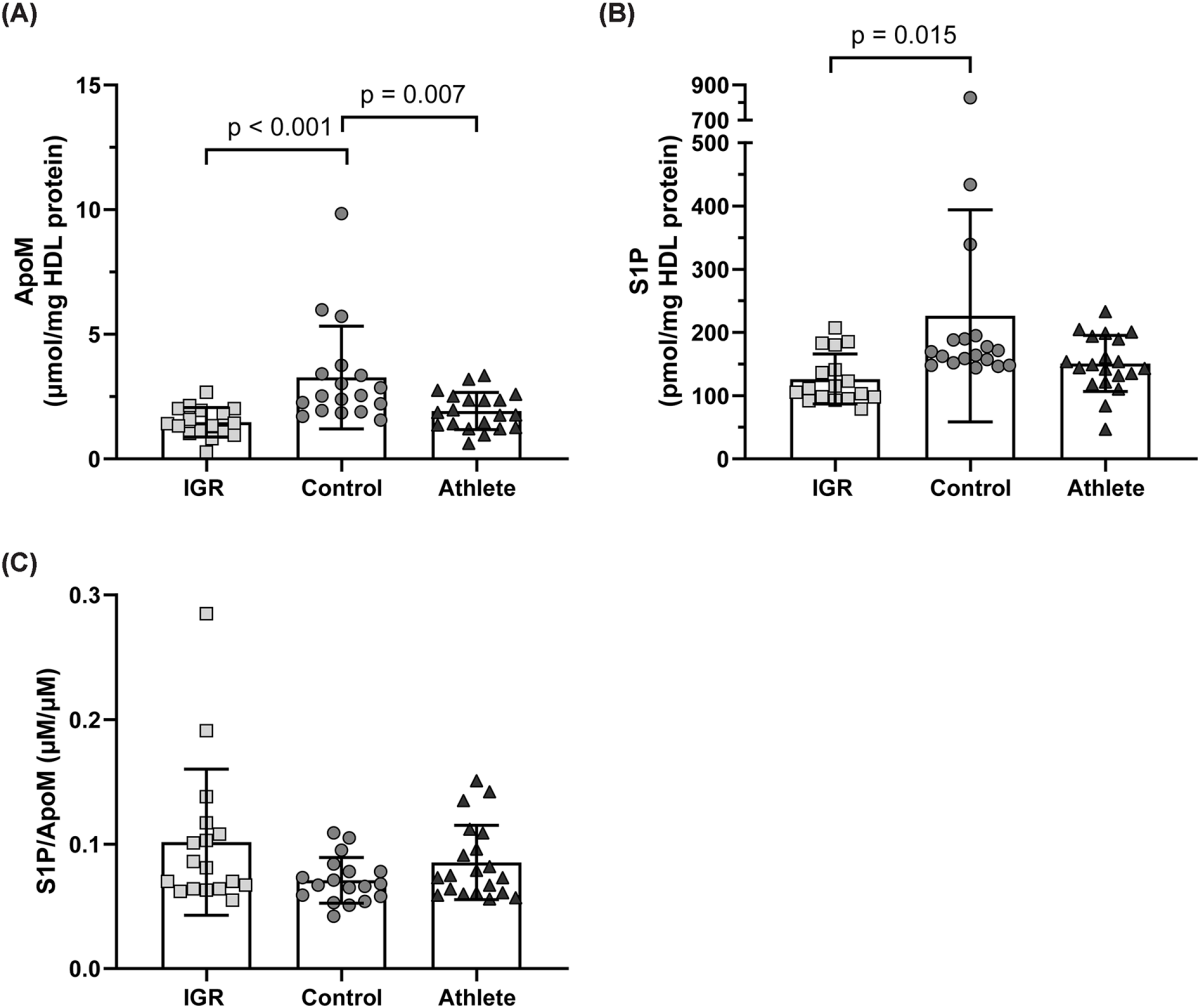
HDL apoM and S1P content in IGR, control, and athletes (**A**) HDL apoM content and (**B**) HDL S1P content, both corrected per mg of HDL protein. (**C**) The ratio of S1P to apoM. In all panels, data expressed as mean ± SD and comparisons made with one-way ANOVA and post-hoc Tukey test. Statistical significance was assumed at *P*<0.05. ApoM, apolipoprotein M; S1P, sphingosine-1-phosphate; IGR, impaired glucose regulation.

The apoM content of HDL was lower in IGR (1.5 ± 0.6 μmol/mg HDL protein, *P*<0.001) and athletes (1.9 ± 0.8 μmol/mg HDL protein, *P*=0.007) compared with controls (3.3 ± 2.1 μmol/mg HDL protein) (Figure 6A). The S1P content of HDL was lower in IGR compared with control (126.3 ± 39.5 pmol/mg HDL and 226.3 ± 167.7 pmol/mg HDL protein respectively, *P*=0.015) (Figure 6B). Though athlete HDL had numerically lower S1P content compared with control (151.0 ± 44.4 pmol/mg HDL protein compared with 226.3 ± 167.7 pmol/mg HDL protein, *P*=0.069), this was not statistically significant. The ratio of S1P to apoM did not significantly differ between the three groups (IGR 0.10 ± 0.06 μmol/μmol; control 0.07 ± 0.02 μmol/μmol; athlete 0.09 ± 0.03 μmol/μmol, ANOVA *P*=0.074) (Figure 6C).

### HDL *in vitro* antioxidant function in athletes, men with IGR and controls

**Figure 7:**
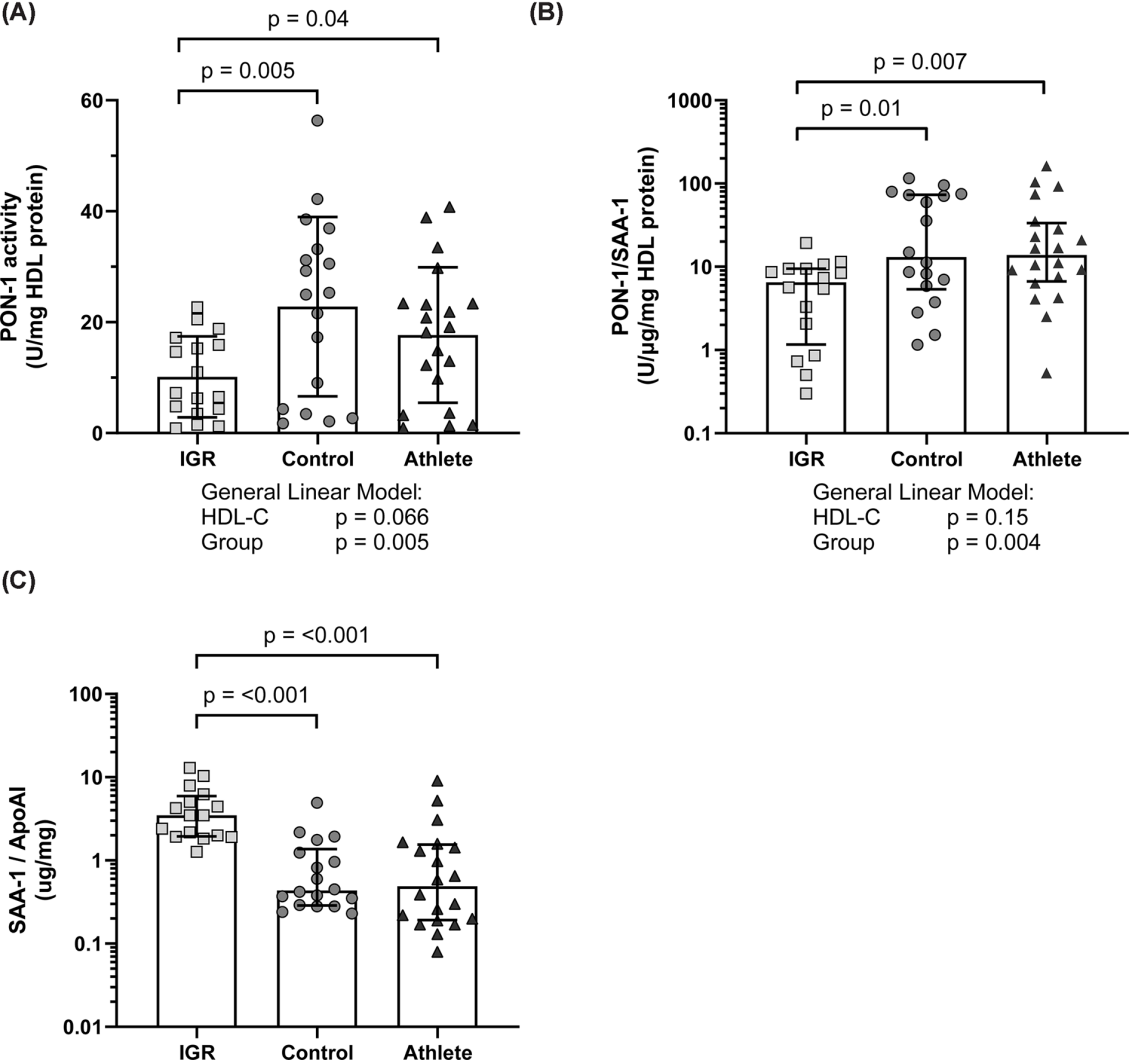
HDL antioxidant capacity in the M-FAT study (**A**) HDL paraoxonase-1 activity corrected to HDL protein content. Data expressed as mean ± SD. Comparisons made with general linear models and post-hoc Tukey test. HDL-C was included as a co-variate. (**B**) The ratio of HDL paraoxonase-1 activity to serum amyloid α-1 content. Data expressed as median ± IQR on a log_10_*y*-axis for data legibility. Comparisons made using log-transformed data with general linear models and post-hoc Tukey test. HDL-C was included as a co-variate. (**C**) The ratio of serum amyloid α-1 to apolipoprotein AI. Data expressed as median ± IQR on a log_10_*y*-axis for data legibility. Comparisons were made with Welch’s ANOVA with post-hoc Dunnett’s T3 test on log-transformed data. Statistical significance was assumed at *P*<0.05. ApoAI, apolipoprotein A-I; HDL-C, high-density lipoprotein cholesterol; IGR, impaired glucose regulation; PON-1, paraoxonase-1; SAA-1, serum amyloid α-1.

PON-1 activity per mg HDL protein was significantly lower in IGR HDL compared with both control (*P*=0.005) and athletes (*P*=0.040, 10.1 ± 7.3 U/mg HDL protein compared with 22.8 ± 16.2 and 17.7 ± 12.2 U/mg HDL protein, respectively) (Figure 7A). This effect was independent of plasma HDL-C. The ratio of HDL PON-1 activity to SAA-1 content was significantly lower in IGR HDL compared with both control (*P*=0.010) and athletes (*P*=0.007, 6.5 [1.2, 9.5] U/μg/mg HDL protein compared with 13.1 [5.4, 73.3] and 13.9 [6.7, 33.6] U/μg/mg HDL protein respectively, median ± IQR) (Figure 7B). This effect was independent of plasma HDL-C. The ratio of SAA-1 to apoA-I content on HDL was significantly higher in the IGR group (3.48 [1.94, 5.92] μg/mg) compared with both control and athlete HDL (0.44 [0.29, 1.37] μg/mg, *P*<0.001 and 0.49 [0.19. 1.55] μg/mg, *P*<0.001, respectively). There was no difference between control and athlete HDL (Figure 7C).

### HDL in vitro anti-inflammatory function in athletes, men with IGR and controls

**Figure 8:**
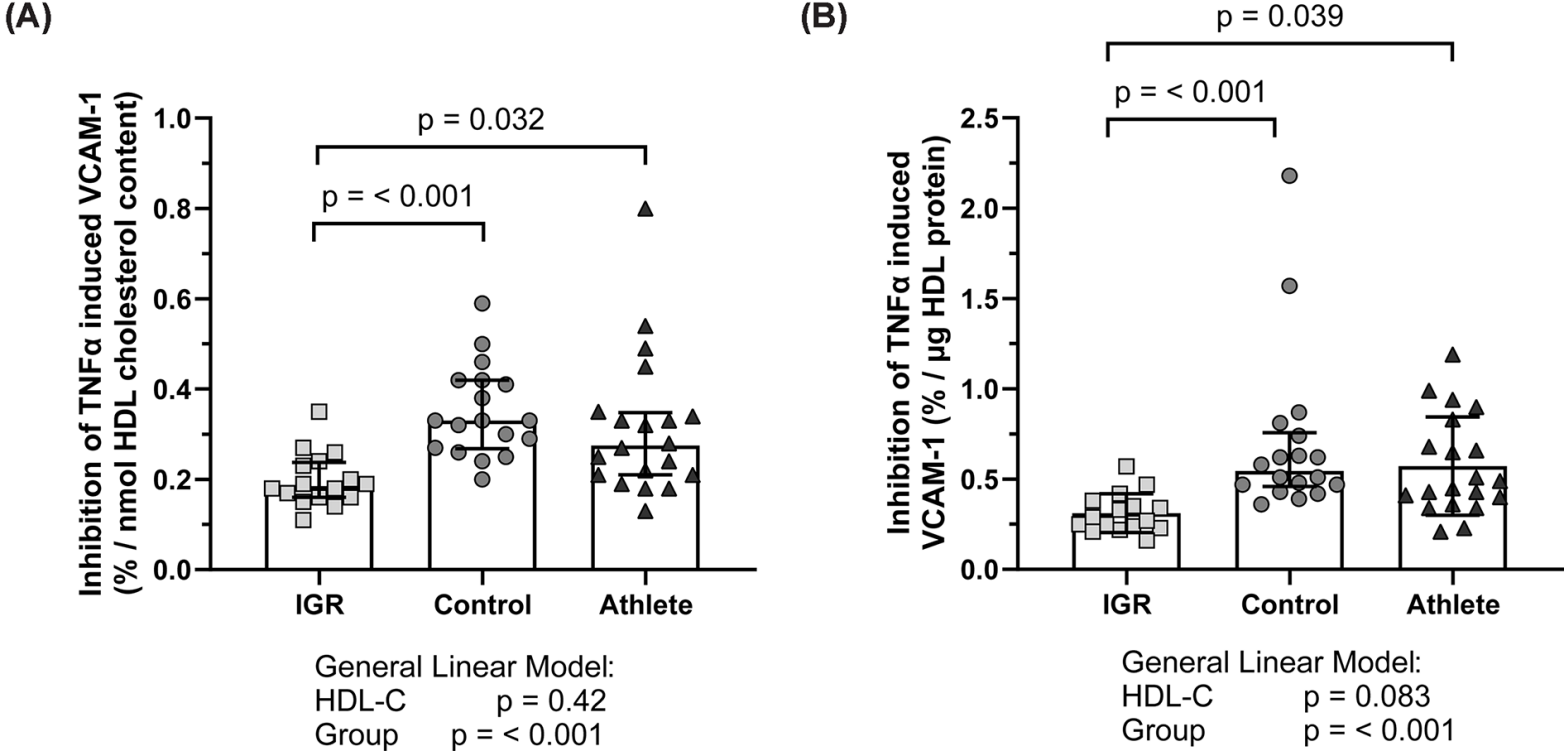
HDL vascular anti-inflammatory function in the M-FAT study (**A**) HDL anti-inflammatory function corrected per nmol HDL cholesterol content and (**B**) corrected per µg HDL protein content. Data expressed as mean ± SD. Comparisons made with general linear models and post-hoc Tukey test. HDL-C was input as a co-variate. Statistical significance was assumed at *P*<0.05. HDL-C, high-density lipoprotein cholesterol; IGR, impaired glucose regulation; TNFα, tumour necrosis factor alpha; VCAM-1, vascular cell adhesion molecule 1.

HDL anti-inflammatory function, when expressed per HDL total cholesterol and per HDL protein, was independent of plasma HDL-C (*P*=0.34 and *P*=0.083, respectively, Figure 8). IGR HDL had significantly lower anti-inflammatory function when corrected for HDL total cholesterol compared with control HDL (0.20 ± 0.06%/nmol compared with 0.35 ± 0.1%/nmol, *P*=0.003, Figure 8A). This pattern continued when correcting HDL anti-inflammatory function for HDL protein; IGR HDL had significantly lower anti-inflammatory function per mg HDL total protein (0.31 ± 0.11%/mg) compared with both control and athlete HDL (0.70 ± 0.46%/mg and 0.57 ± 0.27%/mg, Figure 8B). There was no difference between control and athlete HDL in terms of vascular anti-inflammatory function.

## Discussion

The present study revealed altered HDL protein composition and subclass distribution but not HDL vascular function in middle-aged endurance athletes compared with age-matched healthy sedentary controls. Endurance athletes had larger HDL with an altered HDL proteomic profile characterised by higher apoA-I, apoA-II, apoD, apoF, and transthyretin in an OPLS-DA analysis. Post-hoc univariate analysis demonstrated statistically higher levels of apoD and transthyretin in the athlete group. HDL function, as assessed by PON-1 activity, PON-1/SAA-1 ratio, S1P content, S1P/apoM ratio, and in an anti-inflammatory endothelial cell assay, showed no difference between athletes and controls. As expected, men with IGR showed an altered HDL protein composition and lower function assessed by the afore-mentioned functional assays compared with controls.

The endurance athletes recruited in this study had the lowest body fat percentage, highest fitness (as measured by VO_2_ max) and highest insulin sensitivity (as assessed by the Matsuda index) compared with control and IGR men. Though HOMA_IR_ did not differ between the controls and athletes, this can be explained by the differing nature of the two indices. HOMA_IR_ focuses on the relationship between fasting hepatic glucose production and β-cell insulin secretion to describe baseline insulin resistance, while the Matsuda index of insulin sensitivity describes the whole-body response to a glucose load throughout an oral glucose tolerance test. Plasma HDL cholesterol was not different between athletes and controls, concurring with a meta-analysis of 49 studies which found only a small increase in HDL-C of 2% with exercise [35]. This suggests that the higher HDL apoA-I in athletes is not related to an increase in HDL concentration but rather that their HDL is enriched in apoA-I. HDL total cholesterol was highest in the athlete HDL but was not different between control and IGR HDL, implying that HDL-CEC may be increased with endurance exercise. Direct measurement of HDL-CEC after six-months exercise training in two separate clinical studies showed increased CEC after the intervention [36]. It should be noted that the athletes in the present study had been performing vigorous exercise for a minimum of 5 h per week for 2 years or more at the time of recruitment and that no study to date has measured CEC directly in a similar cohort. The assessment of HDL subclass distribution across the groups found that athletes had the highest proportion of HDL 2b, the largest of the HDL particles, supporting a previous meta-analysis which found that HDL 2 cholesterol increases post aerobic exercise intervention [37].

In terms of the proteomic composition of HDL, apoA-II was higher in athlete HDL compared with IGR HDL, though this difference was not of great magnitude. More interesting was the uniformity of the athlete group compared with the more variable control and IGR groups, suggesting that an exercise-induced increase in apoA-II occurs. This adaptation is likely involved in the increased ABCA1-mediated cholesterol efflux in HDL post-exercise [38] and may contribute to the reduction in neutrophil activity observed post-exercise [39,40]. ApoD, which forms a heterodimer with apo-AII [41], is also increased in athlete HDL compared with control HDL. ApoD is proposed to confer protection from oxidative stress through redox cycling and, at capacity, oligomerisation of oxidised-apoD [42]. Higher apoD in athlete HDL may be part of the adaptation to exercise [43] where short bouts of exercise increase oxidative stress, but repeated exercise such as in this group of athletes provokes a systemic antioxidant adaptation and reduces this effect. Arachidonic acid, a precursor for vaso-active compounds such as prostaglandins, has high affinity for apoD and is known to be stored in lipoproteins including HDL. It may be that apoD can regulate the liberation and storage of arachidonic acid depending on specific physiological stimuli [44]. Prostaglandins are involved in skeletal muscle vasodilation during exercise [45], suggesting a role for HDL in delivering arachidonic acid to muscle in athletes to facilitate prostaglandin synthesis. Transthyretin was higher on athlete HDL compared with control HDL. Exercise increases plasma thyroxine and may therefore also increase plasma levels of its transporter [46]. The detection of transthyretin on HDL may reflect this exercise-induced change.

The vasoactive lipid sphingosine-1-phosphate bound to its carrier apoM has pleiotropic effects on the vascular endothelium including vasodilatory, antioxidant and anti-inflammatory functions [47]. Our finding of lower HDL apoM and lower HDL S1P in men with IGR compared with control conforms with previously performed studies [48,49]. The lower HDL apoM in athletes compared with control was unexpected, given their higher cardiorespiratory fitness and the beneficial effect of aerobic exercise on vascular function (reviewed in [50]). There is evidence that CETP shifts the apoM/S1P complex from HDL onto apoB containing lipoproteins [51] perhaps explaining the lower HDL apoM in athletes, though there is some controversy as to whether CETP activity is higher in athletes, with both no change and an increase seen in the literature [52,53].

Functionally, PON-1 activity was not higher in the athlete HDL compared with control HDL. A recent meta-analysis of studies investigating the effect of exercise on PON-1 found that both acute and chronic exercise had negligible impact on the serum activity of PON-1 [54]. The authors suggest that the lack of increased activity is due to the fact that PON-1 predominantly has effects in the interstitial spaces which would not be detected by a serum measure. As the present study measures HDL PON-1 activity isolated from plasma, the same caveat may apply here. Neither the PON-1/SAA-1 ratio, posited as a marker of overall HDL function [55], nor the SAA-1/apoA-I ratio were different between control and athlete HDL. Together with the lack of difference between control and athlete in terms of the anti-inflammatory function of HDL, we suggest that despite a more favourable composition, athlete HDL does not have improved *in vitro* vascular function. It may be the case that HDL has a ceiling of vascular function, i.e. that healthy HDL function cannot be enhanced. This is in contrast with IGR HDL, which exhibited lower apoA-I content and was characterised by lower apolipoproteins and higher inflammation, coagulation and immune-related protein content in both multivariate and univariate analysis. These changes occurred alongside lower antioxidant and inflammatory function. These findings concur with the wider literature on insulin resistance and HDL composition and function [14,48,56–58], where impaired HDL composition and function can be improved through exercise intervention [16–19]. Our interpretation is that HDL composition reflects the systemic environment but does not always affect HDL function. Insulin resistance is known to be linked to increased systemic inflammation and coagulation. It is therefore likely that the proteins identified on HDL are reflecting the chronic inflammation caused by impaired glucose regulation and insulin resistance rather than any metabolic adaptation to HDL *per se*, and that these proteins may contribute to the reduction in HDL functions. It is likely that these proteins are present on HDL due to their increased plasma abundance. The HDL compositional changes in athletes may reflect the systemic adaptation to an altered metabolic and oxidative environment associated with exercise, though these changes appear not to impact upon HDL function. There is a general consensus that genes linked to HDL concentrations are not associated with adverse cardiovascular outcomes nor are causal in clinical manifestations of CVD, perhaps explaining the failure of HDL-C raising therapy to improve cardiovascular risk [59–62]. The limited differences observed in HDL function despite a more favourable HDL protein composition may reflect this emergent consensus. HDL could be described as a scavenger; its primary purpose is the scavenging of excess peripheral cholesterol for recycling or removal. HDL is therefore in contact with a variety of tissues, cell types and plasma proteins, which likely leads to HDL acquiring new protein and lipid components reflective of the physiological environment. In the same manner, HDL is in contact with other lipoproteins and their remnants, altering HDL protein and lipid composition and therefore also acting as a marker of metabolic function with respect to whole body lipoprotein metabolism. It has been argued that as a static marker of an everchanging metabolic environment, HDL-C is not likely to be causally protective against cardiovascular disease [59]; the findings of this paper propose that HDL protein composition may instead be a marker of the metabolic environment at the time of blood sampling.

The strengths of the present study include clearly differentiated phenotypes in each group studied. The findings in IGR HDL conform with the wider literature therefore validating the novel findings observed in endurance athletes. Endurance athletes are an ‘extreme’ version of cardiometabolic health, where one might have expected to see the largest differences in HDL composition and function against a group of sedentary controls. Our study showed limited compositional and no functional differences between endurance athletes and controls, which therefore suggests that the typically lower levels of exercise in the general healthy population are unlikely to induce compositional and functional changes in HDL. HDL function was assessed *in vitro* using a combination of approaches. Few studies have studied HDL composition and function in supra-healthy men; this study of endurance athletes addresses this research gap. In order to correct for HDL particle number, data were corrected for HDL total protein though correction for HDL total cholesterol was also carried out and showed similar results (data not shown). Furthermore, plasma HDL cholesterol concentration was included as a covariate in the analysis of functional data to correct for differences in circulating HDL levels between the groups. However, the present study did not include measures of HDL cholesterol efflux capacity. The endurance athletes performed different forms of exercise though this information was not collected on recruitment, nor was information pertaining to the participants’ diet. As a cross-sectional study, it was not possible to determine the endurance athletes’ baseline HDL composition and function before their exercise training commenced. Future longitudinal studies may address this limitation. The original study protocol from which these samples were derived did not include an *in vivo* measure of endothelial function as this was not an outcome relevant to its hypothesis. We therefore could not correlate our *in vitro* findings with a physiologically relevant *in vivo* measure, such as brachial artery flow-mediated dilation, in these participants. The proteomics data were expressed as a relative LFQ intensity, and the data-dependent method may mask the detection of lower abundance proteins. There is a limited literature on inflammatory, coagulation and transport proteins and HDL which makes interpretation of their different abundances on athlete, control and IGR HDL difficult. The cell line chosen in this work is reflective of the dermal microvasculature; however, HDL function may be differently affected in cell lines more relevant to exercise / diabetes mellitus such as muscle, kidney or retinal endothelial cells.

The observation that HDL composition reflects systemic and metabolic physiology with limited improvement in antioxidant and anti-inflammatory function has implications for interventions aimed at increasing HDL concentration or improving HDL function in overtly healthy individuals, particularly as those interventions typically also have wider effects on plasma lipids. In insulin resistant individuals, there may be benefits to focussing on improving the systemic and metabolic environment by treating the underlying pathophysiology which may return HDL composition and function back to its physiological normal, rather than focussing on manipulating HDL concentration directly.

## Data Availability

All data used in this article is available on request at [63].

## Abbreviations

Apo: apolipoprotein
BMI: body mass index
CETP: cholesteryl ester transfer protein
CVD: cardiovascular disease
EDTA: ethylenediaminetetraacetic acid
eNOS: endothelial nitric oxide synthase
HbA1c: glycated haemoglobin
HDL: high-density lipoprotein
HDL-C: high-density lipoprotein cholesterol
HDL-CEC: high-density lipoprotein cholesterol efflux capacity
HOMA_IR_: homeostatic model assessment for insulin resistance
IGR: impaired glucose regulation
OGTT: oral glucose tolerance test
PAGE: polyacrylamide gel electrophoresis
PON-1: paraoxonase-1
S1P: sphingosine-1-phosphate
SAA: serum amyloid A
T2DM: Type 2 diabetes mellitus
TNFα: tumour necrosis factor alpha
VCAM-1: vascular cell adhesion molecule 1
